# Artificial Placenta and Artificial Womb Technologies for Lung and Kidney Failure: A Holistic Perspective

**DOI:** 10.1097/MAT.0000000000002443

**Published:** 2025-04-25

**Authors:** Danny J. M. van Galen, Ana Martins Costa, Franziska Siche-Pantel, Ruth Kemper, Niels Rochow, Maria Brandani, Frank R. Halfwerk, Jutta Arens

**Affiliations:** From the * Engineering Organ Support Technologies, Department of Biomechanical Engineering, Faculty of Engineering Technologies, Technical Medical (TechMed) Centre, University of Twente, Enschede, the Netherlands; † Policy and Advocacy Department, European Foundation for the Care of Newborn Infants (EFCNI), Munich, Germany; ‡ Department of Pediatrics, Paracelsus Medical University, Nuremberg, Germany; § Department of Pediatrics, University Medicine Rostock, Rostock, Germany; ¶ Department of Cardio-Thoracic Surgery, Thorax Centrum Twente, Medisch Spectrum Twente, Enschede, the Netherlands.

**Keywords:** artificial womb, artificial placenta, extracorporeal life support, neonatal care, prematurity, respiratory failure, renal failure, ethics

## Abstract

Preterm birth remains the leading cause of mortality among neonates. Despite improvements in neonatal intensive care over the years, current treatments for lung and kidney failure are highly invasive, associated with lifelong disability, and limit family integration. Artificial womb and artificial placenta technologies offer a promising alternative by providing more tailored and less invasive neonatal care. Although these technologies share some similarities, artificial womb and artificial placenta technologies differ significantly in terms of treatment initiation, treatment environment, and the potential to support family-centered care. Moreover, even though acute kidney injury is common in neonatal extracorporeal membrane oxygenation (ECMO) patients, current artificial placenta and artificial womb devices lack renal support functionality. Most artificial womb and artificial placenta studies focus on the technical feasibility of these technologies based on *in-vivo* animal tests. However, translation toward envisioned use of these devices in preterm neonates remains mostly underexposed. A comprehensive stakeholder analysis, including parents and caregivers, is critical to the development of socially acceptable artificial placenta and artificial womb systems. This state-of-the-art review provides an overview of conventional neonatal lung and kidney treatments, delineates the differences between artificial womb and placenta technologies, and addresses the technological and ethical challenges in advancing these technologies toward potential clinical implementation.

Preterm birth is still the leading cause of death among neonates, although a worldwide reduction in neonatal mortality has been observed in recent years.^1^ Annually 2.3 million neonates die with 80% of these deaths occurring in the Sub-Saharan Africa and South Asia regions.^2^ Mortality and morbidity are highest in neonates born extremely preterm, before 28 weeks of gestation, or with extremely low birth weight (<1,000 g).^3,4^ Many of these deaths are caused by immaturity of the lungs, sometimes compounded by kidney failure.^2,5^

Acute kidney injury (AKI) occurs in 8–64% of critically ill preterm neonates.^6–9^ Acute kidney injury is associated with increased morbidity and mortality, especially when used in combination with extracorporeal membrane oxygenation (ECMO).^6,10–12^ Therefore, when taking the natural placenta as a blueprint for device development, toxin removal and fluid management of the fetal blood should not be neglected.

Currently, preterm neonates with pulmonary and/or renal failure are treated with medical devices originally developed for adults and downscaled for neonatal care.^13^ Although treatment with mechanical ventilation, ECMO, and continuous renal replacement therapy (CRRT) may be successfully used in neonates with a body weight over 2.2 kg, these highly invasive therapies do not meet the needs of premature neonates (<34 weeks gestational age).^14,15^ In addition, these high-risk interventions are associated with lifelong disabilities and require multi-interface neonate–machine connections limiting family integration.^16^ Therefore, the perspective on current treatment methodology is changing and research on artificial placenta (AP) and artificial womb (AW) systems has resurged.

The AP has been a research focus since the 1960s for treatment of neonates with severe respiratory failure.^17^ However, development of the AP took a backseat due to improvements in intensive care and development of less complex alternatives including noninvasive respiratory support, mechanical ventilation, surfactant replacement therapy, and antenatal corticosteroid treatment.^18^

Nevertheless, in the past years, development of the AP and AW has evolved through technological innovation. Particularly, with the advent of novel techniques and promising *in-vivo* animal studies showing short-term support for up to 4 weeks, clinical application of AP and AW technology seems closer than ever.^19–23^ This also reignites ethical discussions regarding the use of these technologies.^24,25^

Although both techniques share the aim of mimicking the respiratory functionality of the natural placenta as a bridge to organ maturation, the terms “artificial placenta” and “artificial womb” are easily interchanged and mistakenly confused.

An AP system is envisioned as an oxygenator to which the neonate will be connected for lung support (gas exchange) (Figure 1A).^23,26^ An AW system also contains an oxygenator but submerges the neonate in an artificial amniotic fluid that mimics *in-utero* conditions (Figure 1C).^21^ Alternatively, there is an intermediate strategy in which the neonate is not completely immersed in artificial amniotic fluid. Instead, the neonate is intubated with a tube filled with artificial amniotic fluid (perfluorocarbons) to simulate the fetal environment of the lungs (Figure 1B).^19^ Both fluid-filled lung (FFL) treatment modalities aim to prevent transition from fetal to neonatal physiology. In this way, aeration of the lungs and circulatory changes are postponed with the intention to simulate fetal physiology and support lung maturation. In contrast, an AP system operates in an incubator setting and is initiated after spontaneous breathing of the neonate. All technologies use the umbilical cord vessels for vascular access, with alternatives being the combined pump-driven umbilical and jugular vein configuration.^19^

**Figure 1.**
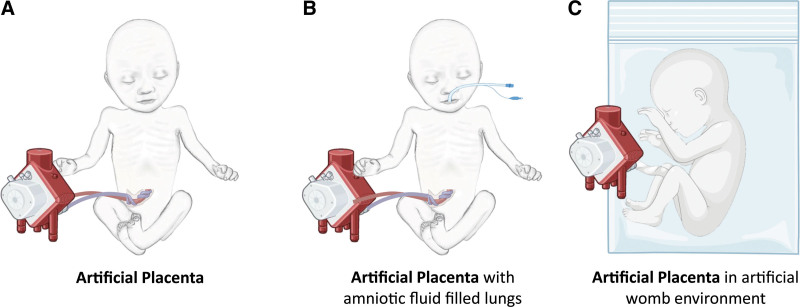
Illustrative overview showing pumpless treatment configurations for a preterm neonate with (**A**) an artificial placenta, (**B**) an artificial placenta with fluid-filled lungs, and (**C**) an artificial womb system. This figure is created with BioRender.com.

So far, AP and AW research has solely focused on the oxygenation function of the placenta. Although AKI occurs in up to 64% of critically ill preterm neonates, to our knowledge, mimicking the renal function of the natural placenta has not yet been integrated into current AP and AW systems.^6^

This multiperspective state-of-the-art review gives insight into treatment methods of preterm neonates with respiratory and/or renal failure, addresses similarities and differences between AP and AW technologies on envisioned human application level, and highlights technical challenges of developing an AP system with combined lung and kidney support. In addition, ethical considerations regarding AW and AP technology are discussed.

## Search Strategy and Selection Criteria

This state-of-the-art review was conducted in accordance with the Preferred Reporting Items for Systematic Reviews and Meta-Analyses (PRISMA) guidelines.^27^ This review includes only literature written in English. Search terms “artificial placenta” AND/OR “artificial womb” were employed in PubMed. Search results were screened based on their titles and abstracts to determine appropriateness for this review. Furthermore, desk research was performed to collect additional relevant literature. The synthesis of content for this review involved a multidisciplinary team of experts, comprising, *e.g*., neonatologists, patient and parent representatives, and engineers.

## Results

### Type of Care per Gestational Age

**Figure 2.**
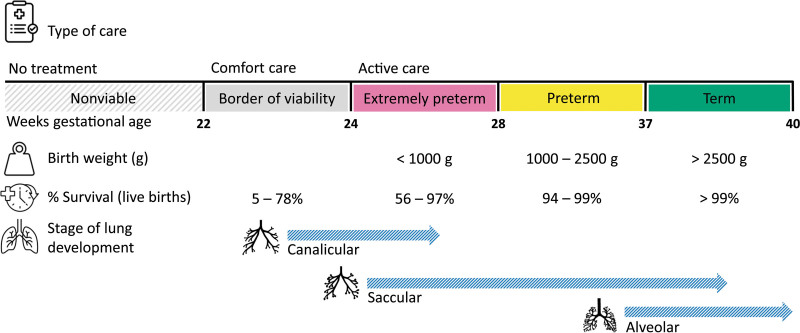
Overview of the different types of treatment applied in preterm neonatal care with regard to gestational age,^30–33^ birth weight,^34^ survival,^35–38^ and stage of lung development.^39^ This figure contains icons from the Noun Project.^40^

Standards and recommendations for when and how to treat neonates vary widely among highly developed countries.^28^ In particular, lower age limits for applying treatment are considered differently between countries. Available resources and sociocultural context mainly influence the variation in guidelines.^29^ Type of care may be divided into withholding care, comfort care with a palliative purpose, or active care to increase survival chances (Figure 2).^30–40^

Current standards generally consider active care for neonates ~24 weeks gestational age.^41^ In practice, active care is in some countries applied even earlier because of clinical conditions of the neonate and parental wishes.^28,41^ Neonates before 22 weeks of gestation with any sign of life are typically provided with comfort (palliative) care. In general, no active treatment is applied for neonates <22 weeks of gestation as the current state of technology fails to provide hope for survival.^28,41^ However, estimating gestational age solely based on the last menstrual period lacks sufficient accuracy and should therefore be complemented by ultrasound examination.^42^

### Respiratory Failure

During intrauterine development, the fetal lung is immersed in amniotic fluid, rendering it nonfunctional for gas exchange. Blood flow originating from the right heart is primarily shunted towards the systemic circulation. This process is facilitated by hypoxic pulmonary vasoconstriction.^43^ Lung development progresses through embryonic, pseudoglandular, canalicular, saccular, and alveolar stages.^39^

A postnatal transition causes significant physiologic adaptations in respiratory and hemodynamic function. This process starts by initiation of breathing at birth and umbilical cord clamping. Lung expansion during the first breath establishes functional residual capacity and enables the onset of pulmonary gas exchange. This event causes a significant reduction in pulmonary vascular resistance, thereby increasing pulmonary blood flow and cardiac venous return.^44^

However, gas exchange can be disturbed by prematurity of the lungs or lung pathology, leading to high morbidity and mortality. In anticipation of preterm birth, antenatal steroids are suggested in preterm neonates with a gestational age of <34 weeks to support surfactant secretion.^45,46^ Surfactant enhances lung compliance and contributes to prevention of respiratory distress syndrome (RDS).^39^ Postnatal treatment includes multiple noninvasive ventilation (NIV) strategies.^47^

**Figure 3.**
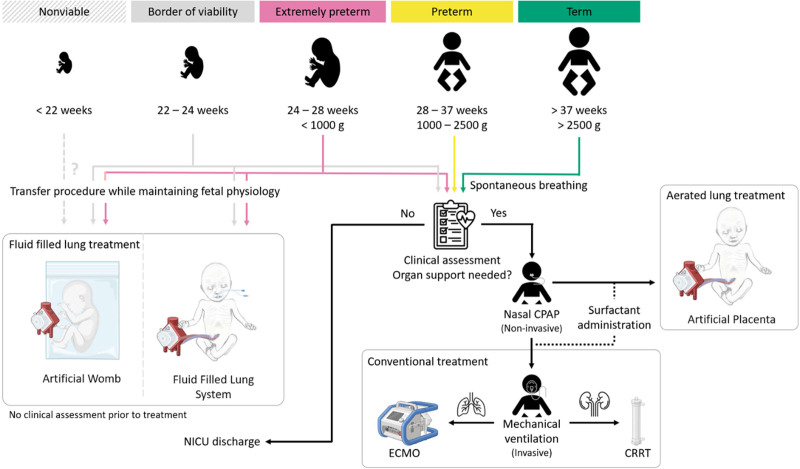
Overview of the envisioned treatment steps of a preterm neonate suffering from severe respiratory and/or renal failure in conventional and future clinical practice. This figure contains icons from the Noun Project^40^ and is created with BioRender.com. CPAP: continuous positive airway pressure; CRRT: continuous renal replacement therapy; ECMO: extracorporeal membrane oxygenation; NICU: neonatal intensive care unit.

Current recommendations suggest that spontaneously breathing preterm neonates should be stabilized with continuous positive air pressure (CPAP) (Figure 3).^46^ In this situation, nasal and nasopharyngeal CPAP could be used.^48^ If apneic or bradycardic, neonates will receive ventilation breaths. Caffeine citrate could be administered in the delivery room to prevent apnea and promote respiratory drive.^49^ It is further suggested that peripheral capillary oxygen saturation (SpO_2_) of 80% or higher (and heart rate >100/min) should be achieved within 5 minutes after birth. Intubation should be reserved for neonates who do not respond to positive pressure ventilation *via* a face mask, nasal prongs, or nasopharyngeal tube.^46^

Extremely preterm neonates <28 weeks of gestation usually receive surfactant. Surfactant is preferably administered by less invasive surfactant application (LISA) or minimally invasive surfactant therapy (MIST) in spontaneously breathing neonates.^46,50^

Ventilation strategies play a crucial role in current clinical management of neonates with RDS. The aim is to optimize gas exchange while minimizing the risk of iatrogenic lung injury. NIV, particularly synchronized nasal intermittent positive pressure ventilation (NIPPV), has shown to be effective in reducing the need for invasive mechanical ventilation (IMV) and improving outcomes.^51^ Noninvasive ventilation modes include high-flow nasal cannula support, nasal CPAP by mechanical ventilators, and bubble CPAP. Recent systematic reviews comparing various modes of NIV for both primary respiratory support and postextubation care concluded that synchronized NIPPV is effective in reducing the necessity for IMV or reintubation in preterm neonates.^52^

Invasive mechanical ventilation should only be used in neonates with RDS when other methods of respiratory support have failed. For neonates requiring IMV, the goal is to maintain “acceptable” blood gases while avoiding both lung overdistension and atelectasis.^53^ Lung-protective modes to prevent bronchopulmonary dysplasia such as volume-targeted ventilation or high-frequency oscillation ventilation should be the first choice for babies with RDS who require IMV.^46,54^

Lung recruitment maneuvers may be used to optimize positive end-expiratory pressure (PEEP), although their impact on outcomes is uncertain.^55^ Once stabilized on IMV, clinicians should promptly plan for weaning to NIV with early extubation encouraged for neonates demonstrating low ventilator settings and adequate spontaneous breathing effort.^56^

When treatment with CPAP and IMV is not sufficient, neonatal ECMO may be considered for late preterm neonates with severe respiratory failure.^57^ Specifically, ECMO may be indicated when weaning from 100% oxygen is not possible after 48 hours of maximal medical treatment.^58^ However, current neonatal ECMO modalities are not suitable for neonates with gestational age <34 weeks and weight <2 kg. In these patients, ECMO treatment is associated with a high risk of intracranial hemorrhage. Therefore, there is a need for a tailored ECMO device that can be safely used in preterm neonates with even lower gestational ages.

### Renal Failure

#### AKI in neonatal patients

Acute kidney injury is defined by an acute decrease in renal function, leading to fluid imbalance, uremia, and altered electrolyte levels. Traditionally, AKI has been defined by a rise in serum creatinine levels above 1.5 mg/dl.^8^ However, since neonates might suffer residual damage even with smaller increases in serum creatinine, a new classification was adopted in 2008 to standardize AKI in neonates using the risk, injury, and failure (RIFLE) criteria.^59^ Similarly, another relevant reference source for the AKI definition, the Kidney Disease Improving Global Outcomes (KDIGO) guideline, has been modified in 2016 to account for particularities of the neonatal patient population.^60^

Acute kidney injury incidence varies from 8% to 64% in critically ill neonates who often already suffer from a number of complications, including hypovolemia, hypotension, ischemia, and inflammation.^6–9^ Neonatal AKI can be treated with renal replacement therapy. However, the indication to start this complex treatment in neonates relies on individual clinical judgement.^61^

Acute kidney injury is a common complication in neonates receiving ECMO. The incidence of AKI in neonatal ECMO patients may be up to 64% with an increased risk of mortality in patients with higher failure classifications.^6^ Several interrelated factors including asphyxia, hypoxia, shock, infection, and hyperinflammation contribute to a high risk of AKI.^6,62^ Moreover, the neonate’s state of respiratory failure leads to hemodynamic instability and reduced renal perfusion. This contributes significantly to the severity of AKI. Unfortunately, exposure to ECMO circuit components aggravates inflammation by increasing levels of endotoxins, exotoxins, and activating leukocytes and thrombocytes.^6^ This can lead to capillary leak syndrome, reduced effective circulating volume, and direct renal injury.^63^ In addition, the mechanical components of the ECMO circuit and the nonphysiologic blood flow conditions can damage the blood cells and can cause hemolysis.^64^ As a result, nephrotoxic-free hemoglobin is released into the plasma and can lead to renal tubular injury.^65^ Additionally, patients on ECMO are at risk for developing thrombosis and bleeding complications. This can impair renal function through direct renal vessel occlusion or hypoperfusion from bleeding.^66^ Furthermore, nephrotoxic medications, such as certain antibiotics, vasopressors, and contrast agents, can directly contribute to kidney injury.

Deteriorated kidney function is associated with an even higher risk of fluid overload in neonatal ECMO. Fluid overload can be defined as a positive net body fluid balance associated with a pathologic state.^67^ This condition affects up to 30% of children and neonates supported by ECMO.^68^ Fluid overload can have negative impact on other organs, especially the lungs.^69^ Characteristics of ECMO treatment, including fluid resuscitation and blood administration to maintain adequate circulation during ECMO, act as a downward spiral to fluid overload. This increases the risk of interstitial edema in the kidneys and may impair renal function, leading to worsening of the patient’s condition.^11,70^

#### Treatment of AKI in neonatal ECMO patients

Continuous renal replacement therapy is typically chosen to treat AKI and fluid overload during ECMO.^70^ Continuous renal replacement therapy allows for the removal of excess fluid and blood toxins for an extended period of time. This continuous treatment with a slower fluid removal rate and continuous solute clearance is tolerated in neonates and minimizes hemodynamic harm to the patient. Most ECMO centers report using some form of CRRT with ECMO in a recent survey.^71^ A majority (85%) of ECMO centers indicate that CRRT with slow continuous ultrafiltration was primarily used to treat and prevent fluid overload in neonatal and pediatric patients.^71^

Typically, CRRT for renal support of neonatal ECMO patients is provided by an additional machine. Additional CRRT support for neonatal ECMO patients can be provided either by independent vascular access with two independent circuits or by connecting the CRRT machine to the ECMO circuit.^70,72^ Contemporary practice use independent circuits of ECMO and CRRT requiring separate pumps, tubing, extracorporeal devices, and cannulae.^71^ Approaches that connect the CRRT system directly to the ECMO circuit can use the same vascular access. However, separate tubing and pumps for the different circuits are still necessary.^70,72,73^ This further exposes the blood to artificial surfaces and unphysiological flow conditions, increasing the risk of blood cell damage, infection, heat loss, and clotting.^70,72–75^ These circuit-related complications can increase the risk of AKI and worsen kidney function in neonates.^6^ Therefore, the development of a single device that integrates both membrane oxygenation and kidney support may offer important benefits to mitigate these risks.^75–77^

### Clinical Treatment Differences Between Artificial Placenta and Artificial Womb

Multiple research groups are working on the development of AP, FFLs, and AW technologies. These systems do not only differ in terms of technical specifications but would also require a different approach in clinical practice. The main clinical differences between these systems are timing of treatment initiation, treatment environment, and the possibility of family-centered care. An overview of research groups working on AP, FFLs, and AW technology and the main features of their systems can be found in Supplement A, Supplemental Digital Content (http://links.lww.com/ASAIO/B485).

#### Care for neonates with an AW

The decision to initiate AW treatment must be made before birth based on the gestational age of the fetus, rather than the assessment of physical functionality. This is particularly relevant for fetuses at the border of viability, as their chance of survival after birth is extremely low with conventional therapies.^37,78^ This is mainly caused by the underdeveloped lung tissue, which prevents the neonate from adequate gas exchange.^79^ However, application of AW treatment for a fetus at >24 weeks gestational age eliminates the option to determine the functionality of the lungs before invasive treatment. This applies also for preterm neonates that might survive when only supported by NIV strategies.^80^ Therefore, application of AW treatment may lead to overtreatment in this gestational age group and is therefore worthy of discussion.

Most research groups use C-section as the delivery mode for AW treatment. The fetus needs to be transferred to the artificial amniotic environment before the spontaneous breathing process starts. This means that the fetal circulation is maintained and that anatomical and physiologic circulation changes are postponed. Although the chance of spontaneous breathing is equal between C-section and natural birth, the advantage of C-section may be that every procedural step can be scheduled. Researchers also investigate ways to transfer the fetus into a bag filled with artificial amniotic fluid under natural birth conditions avoiding spontaneous breathing.^81^

Skin-to-skin care improves the neonate’s and caregiver’s (mental) health and increases breastfeeding rates. However, skin-to-skin care would not be accessible through an AW.^82–85^ Moreover, it is yet unknown what long-term consequences the early separation of the neonate from another living organism might have on its development. Naturally constant stimuli such as movement, sound, maternal antibodies, or exposure to language remain unavailable in an AW. Mechanisms, describing the interaction between external stimuli and the plasticity of the central nervous system early in life are not well understood yet and become more important when considering severely stressful stimuli like pain, that might occur during treatment. Therefore, some groups working on AW technology plan to incorporate some stimuli into their systems, such as movement and sounds recorded from the mother.^86^

#### Care for neonates with an AP

A premature neonate can be delivered either naturally or by C-section even if an AP system is expected to be needed. Spontaneous breathing is stimulated and the umbilical cord is cut. This process is followed by a cascade of physiologic changes from fetal to *ex-utero* life with normal circulation.^44^ Therefore, unlike AW treatment, AP treatment would allow for assessment of the physical functionality of the lungs and kidneys to determine if and what type of treatment is needed. This may contribute to prevention of overtreatment and may even limit the risk on treatment-related morbidity for both mother and neonate.

Vascular access through the umbilical cord is a limiting factor for the applicability of AP treatment. Postnatal degeneration of the umbilical cord tissue must be prevented to potentially use umbilical vessels for device connection until the neonatologist instigates AP treatment. Furthermore, supporting a neonate with an AP would allow for family-centered care as the neonate will be placed in an incubator environment. This would enable the parents to touch their neonate and, in the most favorable situation, interact with and hold their child if the neonate’s clinical situation allows.

### Ethical and Parental Considerations in Artificial Placenta and Artificial Womb Technology

Artificial placenta and AW technologies are developed to treat neonates at the border of viability. This requires mutual decision-making from parents and the medical team. The device design of these technologies plays an important role in this intensive period of critical care involving both medical staff and parents.

Therefore, development and implementation of AW and placenta devices should include the multiprofessional expertise of engineers, physicians, nursing staff, and nonprofessional caregivers.^87^ However, currently the novelty of both neonatal devices is paralleled by lacking ethical guidelines in the fields of societal dialogs, value-sensitive technological design, or human research ethics. These guidelines are needed on the way to an incontrovertible moral and legal framework.^87^

At the basis of all medical interventions stand the ideas of respect for autonomy, beneficence, nonmaleficence, and justice. Therefore, one of the central questions for both technologies involves the legally and medically correct term for the treated patient. Depending on whether the patient is referred to as a neonate or a fetus, their access to AP or womb technologies, permissible withdrawal from them, and parental rights will be affected.^25^ Although AW technology might be more suitable for preterm situations at the border of viability, where it might even be questioned whether the patient should be considered born, an AP is being developed to treat patients who are clearly considered neonates. Therefore, they have the right to care, freedom from discrimination and unnecessary pain as stated in the UN Convention on the Rights of the Child.^88^

This also questions the applicability of these technologies to the intended target populations. The use of global neonatal mortality statistics to emphasize the potential impact of these technologies should be adjusted to reflect the likelihood of their availability in low-resource settings that lack modern healthcare facilities. As long as these technologies cannot be designed for use in a low-resource setting, global neonatal mortality rates do not accurately represent the target population for AP and AW technologies.

Proper terminology and framing are identified as important for the reception of such technologies. For example, mainstream media channels reach nonprofessional audiences and should use easy-to-understand language. For them, the term “artificial womb” may suggest it can do more than it actually can. It plays with the idea of providing a fully functioning environment outside the mother for a neonate to grow and mature. It may suggest that other natural processes or even the human being as the carrier of the fetus can be replaced. Rather, the primary goal of these new technologies should be portrayed as an effort to replace current invasive treatments that are used at the edge of viability but do not replace full pregnancy in an artificial way.^87^ The parallel discussions of complete gestation outside the human womb are in that sense misleading and do not represent the idea behind AP and AW technologies.^89^

#### Treatment choice and impact for parents, health care providers, and society

Exposure to AP or AW technology may result in different acute and long-term health outcomes for a neonate. This raises the question of whether parameters such as survival correlate with long-term effects on later quality of life. A thorough health technology assessment, including cost–benefit analysis, could provide insight into the impact of these technologies on society. Treatment decisions affect not only the neonatal patient but also the quality of life and perceptions of parents, loved ones, and caregivers. Their mental health may be affected differently by having to deal with one or the other treatment option. An AW, for example, may be received as an unapproachable machine that would prohibit direct physical interaction between caregiver and neonate. A portable device, on the other hand, seems to allow for a more family-centered approach to care. In this context, it becomes even more important to incorporate the values of parents and caregivers into complex, ethically challenging, and emotionally taxing pediatric and perinatal decisions.^90,91^

#### Inclusion of patients, parents, and caregivers in clinical research

Although both technologies have unique ethical challenges, they also face similar issues when it comes to involving parents and caregivers in clinical trials.^24,92^ Safe medical device development is pivotal, where clinical trials involving neonates raise complex ethical considerations. Participants themselves are not able to consent to their participation in a trial. Instead, their parents are asked to make a deliberate decision in an often highly vulnerable and emotional situation, like during or immediately after an unexpected preterm birth. Clinical trials with AP or AW technology will only be performed in life-threatening situations for the neonate when all other conservative treatment alternatives are ineffective. To address these challenges, the parents’ unsolicitous, informed consent to all procedures is mandatory. This can be achieved by providing timely and understandable information in a calm environment, through a trusting relationship with the health care provider in charge. At the same time, a continuous consent process that reaffirms the consent of the parents at multiple points during a treatment or clinical trial is essential.^93–95^

The needs and rights of parents and neonates can furthermore be included by consulting patient representatives. In EU-funded projects, this approach is highly recommended for all clinical research and even mandatory for pharmacological research. By gathering a network of experienced stakeholders, patient representative organizations provide information regarding the needs and rights of preterm babies and their families during all stages of a device development to ensure that the voice of patients, parents, and caregivers is heard. A patient-involving conceptualization of new medical devices not only enhance its safety, usability, and sustainability but may simultaneously encourage societal acceptance and understanding. Thereby, some of the complex ethical challenges associated with developing life-sustaining devices for this vulnerable patient population can be addressed proactively.

### Developmental Challenges of an Artificial Placenta System

Although system requirements for the AP are applicable for aerated lung, FFL, and AW environments, this section will focus on the development of the AP for use in aerated conditions.

The development of an ideally pumpless AP with integrated kidney support should realize a less complex and less invasive treatment modality that enables family-centered care. It should allow for a more compact device design with fewer cannulas, less tubing, and minimized artificial surfaces compared with current treatment alternatives.^75^ Nevertheless, the development of such a device faces major challenges.

**Figure 4.**
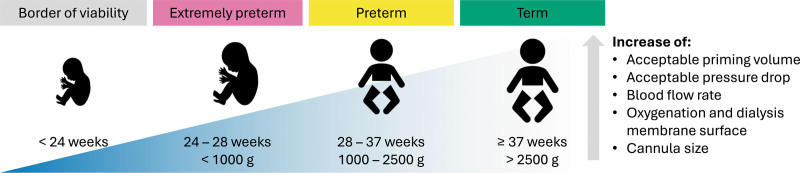
Overview of changing parameters related to neonatal growth affecting device design of the artificial placenta. This figure contains icons from the Noun Project.^40^

An AP should be suitable for a wide range of (preterm) neonates (~24–40 weeks gestational age) with a wide variety of body weights (~400–5,000 g). Age and size of the neonate will affect several important device design parameters, including maximum priming volume and maximum allowable pressure drop (Figure 4). Priming volume should be minimized to avoid the need for (placental) blood transfusion. Pressure drop must be limited when extracorporeal blood flow is driven solely by the neonatal heart. Unfortunately, these two design parameters are opposing each other. Therefore, a low resistance blood flow path with as low as possible priming volume requires a thorough, well-balanced design of the extracorporeal circuit. As a certain membrane area is physically needed, inlet and outlet geometries of the device play a crucial role here.

As the neonate grows over time, higher flow rates, larger oxygenation and dialysis membrane surfaces, and larger cannulas are required. Therefore, a device that can adapt to the neonate’s needs is desirable. A modular system that allows expansion and replacement of certain parts of the system during ongoing treatment may address these needs. Previous studies have described the development of a modular gas exchange area adjustable oxygenator design and a stackable microfluidic device.^20,96^ Another recent development showed an expandable womb system that can adapt its volume to fetal growth. Similar modular design principles may also be beneficial for longer-term AP treatment.

The use of systemic anticoagulation in preterm neonates is associated with a high risk of brain hemorrhage.^97^ Therefore, the AP system must be designed in such a way that the need for systemic anticoagulation is avoided. Alternatively or additionally, antithrombogenic agents could be used in device coatings or in the sweep gas (*e.g*., nitric oxide) to prevent blood clotting in the system.^98^

The umbilical cord vessels of the neonate can be used to connect the neonate to the AP. This prevents peripheral or central cannulation in vulnerable preterm neonates. At the same time, these access lines could also be used to provide nutrition and additional drugs. Degeneration of the umbilical stump after cord clamping must be prevented to maintain the connection throughout the duration of treatment. Therefore, the environment surrounding the umbilical stump could be designed to replicate the fluidic environment present *in utero*, as is done in the AW. Moreover, degeneration of the umbilical vessels after the umbilical clamping procedure should be prevented. Both the umbilical arteries and umbilical vein should be kept accessible until it has been decided whether AP treatment is needed. If treatment is required, the neonate must be attached to the AP system and umbilical perfusion must be resumed. Previous publications have described the use of vasodilator drugs (*e.g*., papaverine) to improve the umbilical cannulation procedure.^19,21,99^ An alternative strategy may be to temporarily keep the vessels mechanically open.

Although umbilical vessel catheterization is common, umbilical vein and artery cannulation with larger bore (10–14 Fr) cannulas has not been established. Therefore, a high-fidelity neonatal cannulation simulator based on human anatomy and physiology may be useful. Such a device could be used for *in-vitro* testing of new umbilical cannula designs, as well as a training module for clinical staff to simulate the umbilical cord cannulation procedure. Likewise, a simulator to train the neonatal transfer procedure is being developed for the AW system.^81^

In addition, high-quality *in-vitro* testing is important to minimize *in-vivo* testing resources in early device development of an AP system. Therefore, blood testing in accordance with ISO 7199:2016 (oxygenator testing), ISO 8637:2017 (hemodialyzer testing), and ISO 18193:2021 (cannula testing) standards is essential to verify device performance and safety.

## Conclusions

Although AP and AW technologies are designed to provide respiratory support, differences in treatment initiation, treatment environment, and ability to provide family-centered care characterize the unique features of both technologies. Critical evaluation of patient characteristics is required to determine which treatment modality is most appropriate for a given clinical scenario.

Although the development of the AP and AW still faces serious challenges, human clinical trials are on the horizon. Continuation of the debate on the development and use of these technologies should guide the technology toward ethical and social acceptance.

The limitations of conventional neonatal treatment emphasize the need for a device that combines pulmonary and renal support. However, current AP and AW systems do not include this functionality. Therefore, the development of an AP device with integrated lung and kidney support could represent a pivotal advance in family-centered intensive care for critically ill preterm neonates.

## Acknowledgments

Illustrations in this article were partly created using icons from the Noun Project and BioRender.com.

Members of ArtPlac Research Consortium: Niels Rochow, Christoph Fusch, Maria Brandani, Andrea Krapf, Kelly Payne, Jutta Arens, Danny van Galen, Dimitrios Stamatialis, Lucia Romano, Odyl ter Beek, Lisa Prahl Wittberg, Imane El Jirari, Cian Murphy, Eamonn Stenson, Aine O’Grady, Ronan Scott, Peter Ewert, Stanimir Georgiev, Katarzyna Gendera, Daan Ophelders, Tim Wolfs, Thorsten Knoll, Ulrich Haag, Ruth Kemper, Helmut Hummler, John Brash, Kyla Sask, Ravi Selvaganapathy, Gerhard Fusch, Iva Pashkuleva, Stefan Eckstein.

## Supplementary Material

## References

[1] Cao G Liu J Liu M: Global, regional, and national incidence and mortality of neonatal preterm birth, 1990-2019. JAMA Pediatr. 176: 787–796, 2022. 35639401 10.1001/jamapediatrics.2022.1622 PMC9157382

[2] Sharrow D Hug L Liu Y Lindt N Nie W You D: Levels & Trends in Child Mortality—Report 2023—Estimates developed by the United Nations Inter-agency Group for Child Mortality Estimation. UNICEF, 2024.

[3] Seri I Evans J: Limits of viability: definition of the gray zone. J Perinatol. 28: S4–S8, 2008. 18446176 10.1038/jp.2008.42

[4] Lui K Bajuk B Foster K: Perinatal care at the borderlines of viability: A consensus statement based on a NSW and ACT consensus workshop. Med J Aust. 185: 495–500, 2006. 17137454 10.5694/j.1326-5377.2006.tb00664.x

[5] Nada A Bonachea EM Askenazi DJ: Acute kidney injury in the fetus and neonate. Semin Fetal Neonatal Med. 22: 90–97, 2017. 28034548 10.1016/j.siny.2016.12.001 PMC5373985

[6] Zwiers AJ de Wildt SN Hop WC: Acute kidney injury is a frequent complication in critically ill neonates receiving extracorporeal membrane oxygenation: A 14-year cohort study. Crit Care. 17: R151, 2013. 23883698 10.1186/cc12830 PMC4057448

[7] Gallo D de Bijl-Marcus KA Alderliesten T Lilien M Groenendaal F: Early acute kidney injury in preterm and term neonates: Incidence, outcome, and associated clinical features. Neonatology. 118: 174–179, 2021. 33780939 10.1159/000513666

[8] Selewski DT Charlton JR Jetton JG: Neonatal acute kidney injury. Pediatrics. 136: e463–e473, 2015. 26169430 10.1542/peds.2014-3819

[9] Hu Q Li S-J Chen Q-L Chen H Li Q Wang M: Risk factors for acute kidney injury in critically Ill neonates: A systematic review and meta-analysis. Front Pediatr. 9: 666507, 2021. 34336736 10.3389/fped.2021.666507 PMC8316634

[10] Lazarovits G Ofek Shlomai N Kheir R Bdolah Abram T Eventov Friedman S Volovelsky O: Acute kidney injury in very low birth weight infants: A major morbidity and mortality risk factor. Children (Basel). 10: 242, 2023. 36832371 10.3390/children10020242 PMC9955621

[11] Murphy HJ Gien J Sahay R: Acute kidney injury, fluid overload, and renal replacement therapy differ by underlying diagnosis in neonatal extracorporeal support and impact mortality disparately. Blood Purif. 50: 808–817, 2021. 33461205 10.1159/000512538

[12] Jetton JG Boohaker LJ Sethi SK; Neonatal Kidney Collaborative (NKC): Incidence and outcomes of neonatal acute kidney injury (AWAKEN): A multicentre, multinational, observational cohort study. Lancet Child Adolesc Health. 1: 184–194, 2017. 29732396 10.1016/S2352-4642(17)30069-X PMC5933049

[13] Fuerch JH Sanderson P Barshi I Liley H: Developing safe devices for neonatal care. Semin Perinatol. 43: 151176, 2019. 31662216 10.1053/j.semperi.2019.08.005

[14] Hwang TJ Kesselheim AS Bourgeois FT: Postmarketing trials and pediatric device approvals. Pediatrics. 133: e1197–e1202, 2014. 24733871 10.1542/peds.2013-3348 PMC4531281

[15] Section On Cardiology And Cardiac SurgerySection On Orthopaedics: Off-label use of medical devices in children. Pediatrics. 139: e20163439, 2017. 28025239 10.1542/peds.2016-3439

[16] Amodeo I Di Nardo M Raffaeli G: Neonatal respiratory and cardiac ECMO in Europe. Eur J Pediatr. 180: 1675–1692, 2021. 33547504 10.1007/s00431-020-03898-9 PMC7864623

[17] Schoberer M Arens J Lohr A: Fifty years of work on the artificial placenta: Milestones in the history of extracorporeal support of the premature newborn. Artif Organs. 36: 512–516, 2012. 22309513 10.1111/j.1525-1594.2011.01404.x

[18] Schwartz RM Luby AM Scanlon JW Kellogg RJ: Effect of surfactant on morbidity, mortality, and resource use in newborn infants weighing 500 to 1500 g. N Engl J Med. 330: 1476–1480, 1994. 8164699 10.1056/NEJM199405263302102

[19] Kading JC Langley MW Lautner G: Tidal flow perfusion for the artificial placenta: A paradigm shift. ASAIO J. 66: 796–802, 2020. 31577624 10.1097/MAT.0000000000001077 PMC7103518

[20] Dabaghi M Rochow N Saraei N: Miniaturization of artificial lungs toward portability. Adv Mater Technol. 5: 2000136, 2020

[21] Larson AC De Bie FR Chang J Davey MG Flake AW: The EXTrauterine environment for neonatal development: Present and future. Pediatr Dev Pathol. 25: 253–262, 2022. 35102770 10.1177/10935266211067110

[22] Usuda H Watanabe S Saito M: Successful use of an artificial placenta-based life support system to treat extremely preterm ovine fetuses compromised by intrauterine inflammation. Am J Obstet Gynecol. 223: 755.e1–755.e20, 2020. 10.1016/j.ajog.2020.04.036 32380175

[23] Arens J Schoberer M Lohr A: NeonatOx: A pumpless extracorporeal lung support for premature neonates. Artif Organs. 35: 997–1001, 2011. 21995519 10.1111/j.1525-1594.2011.01324.x

[24] Kukora SK Mychaliska GB Weiss EM: Ethical challenges in first-in-human trials of the artificial placenta and artificial womb: Not all technologies are created equally, ethically. J Perinatol. 43: 1337–1342, 2023. 37400494 10.1038/s41372-023-01713-5

[25] Werner KM Mercurio MR: Ethical considerations in the use of artificial womb/placenta technology. Semin Perinatol. 46: 151521, 2022. 34893338 10.1016/j.semperi.2021.151521

[26] Schoberer M Arens J Erben A: Miniaturization: The clue to clinical application of the artificial placenta. Artif Organs. 38: 208–214, 2014. 24147856 10.1111/aor.12146

[27] Page MJ McKenzie JE Bossuyt PM: The PRISMA 2020 statement: An updated guideline for reporting systematic reviews. BMJ. 372: n71, 2021. 33782057 10.1136/bmj.n71 PMC8005924

[28] Guillen U Weiss EM Munson D: Guidelines for the management of extremely premature deliveries: A systematic review. Pediatrics. 136: 343–350, 2015. 26169424 10.1542/peds.2015-0542

[29] De Proost L Verweij EJT Ismaili M’hamdi H: The edge of perinatal viability: Understanding the Dutch position. Front Pediatr. 9: 634290, 2021. 33598441 10.3389/fped.2021.634290 PMC7882530

[30] Moutquin JM: Classification and heterogeneity of preterm birth. BJOG. 110: 30–33, 2003. 12763108 10.1016/s1470-0328(03)00021-1

[31] Medicine TACoOaGCoOPSfM-F: ACOG committee opinion no 579: Definition of term pregnancy. Obstet Gynecol. 122: 1139–1140, 2013. 24150030 10.1097/01.AOG.0000437385.88715.4a

[32] Fleischman AR Oinuma M Clark SL: Rethinking the definition of “term pregnancy.” Obstet Gynecol. 116: 136–139, 2010. 20567179 10.1097/AOG.0b013e3181e24f28

[33] World Health Organization: WHO recommendations for care of the preterm or low-birth-weight infant, 2022. 36449655

[34] Dobbins TA Sullivan EA Roberts CL Simpson JM: Australian national birthweight percentiles by sex and gestational age, 1998–2007. Med J Aust. 198: 189–189, 2012. 10.5694/mja11.11331 22938128

[35] Network CN: Annual report 2022, 2022.

[36] Mercer BM: Periviable birth and the shifting limit of viability. Clin Perinatol. 44: 283–286, 2017. 28477660 10.1016/j.clp.2017.02.002

[37] Berger TM Bernet V El Alama S: Perinatal care at the limit of viability between 22 and 26 completed weeks of gestation in Switzerland. 2011 revision of the Swiss recommendations. Swiss Med Wkly. 141: w13280, 2011. 22009720 10.4414/smw.2011.13280

[38] Perined: Perined dataset tool. Available at: https://www.peristat.nl/.

[39] Nikolić MZ Sun D Rawlins EL: Human lung development: Recent progress and new challenges. Development. 145: dev163485, 2018. 30111617 10.1242/dev.163485 PMC6124546

[40] Noun Project. Icons from Noun Project under CC-BY license. Muhammad Ikhsan (“medical checklist”) is, Suncheli Project (“weight”), M. Oki Orlando (“nasal cannula”), Dahlia nur aini (“kidneys”), Suncheli Project (“human lungs”), wildan putra (“oxygen mask”), Chimol (“medical checklist”), Gan Khoon Lay (“baby”), Milinda Courey (“fetus”), The Noun Project, 2024.

[41] Pignotti MS Donzelli G: Perinatal care at the threshold of viability: An international comparison of practical guidelines for the treatment of extremely preterm births. Pediatrics. 121: e193–e198, 2008. 18166538 10.1542/peds.2007-0513

[42] Practice CoO: Committee opinion No 700:methods for estimating the due date. Obstet Gynecol. 129: e150–e154, 2017. 28426621 10.1097/AOG.0000000000002046

[43] Dunham-Snary KJ Wu D Sykes EA: Hypoxic pulmonary vasoconstriction: From molecular mechanisms to medicine. Chest. 151: 181–192, 2017. 27645688 10.1016/j.chest.2016.09.001 PMC5310129

[44] Hillman N Kallapur SG Jobe A: Physiology of transition from intrauterine to extrauterine life. Clin Perinatol. 39: 769–783, 2012. 23164177 10.1016/j.clp.2012.09.009 PMC3504352

[45] Lemyre B Moore G: Counselling and management for anticipated extremely preterm birth. Paediatr Child Health. 22: 334–341, 2017. 29485138 10.1093/pch/pxx058 PMC5804811

[46] Sweet DG Carnielli VP Greisen G: European consensus guidelines on the management of respiratory distress syndrome: 2022 update. Neonatology. 120: 3–23, 2023. 36863329 10.1159/000528914 PMC10064400

[47] Gnanaratnem J Finer NN: Neonatal acute respiratory failure. Curr Opin Pediatr. 12: 227–232, 2000. 10836158 10.1097/00008480-200006000-00009

[48] Kurz H: Influence of nasopharyngeal CPAP on breathing pattern and incidence of apnoeas in preterm infants. Biol Neonate. 76: 129–133, 1999. 10460950 10.1159/000014152

[49] Dani C Cecchi A Ciarcià M: Enteral and parenteral treatment with caffeine for preterm infants in the delivery room: A randomised trial. Paediatr Drugs. 25: 79–86, 2023. 36301511 10.1007/s40272-022-00541-y PMC9810558

[50] Ng EH Shah V: Guidelines for surfactant replacement therapy in neonates. Paediatr Child Health. 26: 35–49, 2021. 33552321 10.1093/pch/pxaa116 PMC7850281

[51] Dargaville PA Gerber A Johansson S; Australian and New Zealand Neonatal Network: Incidence and outcome of CPAP failure in preterm infants. Pediatrics. 138: e20153985, 2016. 27365307 10.1542/peds.2015-3985

[52] Ramaswamy VV Bandyopadhyay T Nanda D: Efficacy of noninvasive respiratory support modes as postextubation respiratory support in preterm neonates: A systematic review and network meta-analysis. Pediatr Pulmonol. 55: 2924–2939, 2020. 32757365 10.1002/ppul.25007

[53] Moretti C Gizzi C: Synchronized nasal intermittent positive pressure ventilation. Clin Perinatol. 48: 745–759, 2021. 34774207 10.1016/j.clp.2021.07.005

[54] Chakkarapani AA Adappa R Mohammad Ali SK: “Current concepts in assisted mechanical ventilation in the neonate”- part 2: Understanding various modes of mechanical ventilation and recommendations for individualized disease-based approach in neonates. Int J Pediatr Adolesc Med. 7: 201–208, 2020. 33319021 10.1016/j.ijpam.2020.11.002 PMC7729247

[55] Blazek EV East CE Jauncey-Cooke J Bogossian F Grant CA Hough J: Lung recruitment manoeuvres for reducing mortality and respiratory morbidity in mechanically ventilated neonates. Cochrane Database Syst Rev. 3: CD009969, 2021. 33781001 10.1002/14651858.CD009969.pub2 PMC8094588

[56] Ferguson KN Roberts CT Manley BJ Davis PG: Interventions to improve rates of successful extubation in preterm infants: A systematic review and meta-analysis. JAMA pediatr. 171: 165–174, 2017. 27918754 10.1001/jamapediatrics.2016.3015

[57] Brogan TV Lequier L Lorusso R MacLaren G Peek G (eds): Extracorporeal Life Support: the ELSO Red Book, Ann Arbor, MI, Extracorporeal Life Support Organization, 2017.

[58] ELSO: ELSO Guidelines for Cardiopulmonary Extracorporeal Life Support. Guidelines for Neonatal Respiratory Failure, Extracorporeal Life Support Organization, Version 1.4, 2017.

[59] Akcan-Arikan A Zappitelli M Loftis LL Washburn KK Jefferson LS Goldstein SL: Modified RIFLE criteria in critically ill children with acute kidney injury. Kidney Int. 71: 1028–1035, 2007. 17396113 10.1038/sj.ki.5002231

[60] Khwaja A: KDIGO clinical practice guidelines for acute kidney injury. Nephron Clin Pract. 120: c179–c184, 2012. 22890468 10.1159/000339789

[61] Spector BL Misurac JM: Renal replacement therapy in neonates. Neoreviews. 20: e697–e710, 2019. 31792157 10.1542/neo.20-12-e697

[62] Kupferman JC Yitayew M Rastogi S: Acute kidney injury in term neonates. Curr Treat Options Pediatr. 4: 386–403, 2018.

[63] Millar JE Fanning JP McDonald CI McAuley DF Fraser JF: The inflammatory response to extracorporeal membrane oxygenation (ECMO): A review of the pathophysiology. Crit Care. 20: 387, 2016. 27890016 10.1186/s13054-016-1570-4 PMC5125043

[64] Ostermann M Lumlertgul N: Acute kidney injury in ECMO patients. Crit Care. 25: 313, 2021. 34461966 10.1186/s13054-021-03676-5 PMC8405346

[65] Garg M: Intravascular hemolysis and complications during extracorporeal membrane oxygenation. NeoReviews. 21: e728–e740, 2020. 33139510 10.1542/neo.21-11-e728

[66] Zeibi Shirejini S Carberry J McQuilten ZK Burrell AJC Gregory SD Hagemeyer CE: Current and future strategies to monitor and manage coagulation in ECMO patients. Thromb J. 21: 11, 2023. 36703184 10.1186/s12959-023-00452-z PMC9878987

[67] Goldstein SL Akcan-Arikan A Alobaidi R; Pediatric ADQI Collaborative: Consensus-based recommendations on priority activities to address acute kidney injury in children: A modified Delphi consensus statement. JAMA Netw Open. 5: e2229442–e2229442, 2022. 36178697 10.1001/jamanetworkopen.2022.29442 PMC9756303

[68] Selewski DT Askenazi DJ Bridges BC: The impact of fluid overload on outcomes in children treated with extracorporeal membrane oxygenation: A multicenter retrospective cohort study. Pediatr Crit Care Med. 18: 1126–1135, 2017. 28937504 10.1097/PCC.0000000000001349 PMC5716912

[69] Rutledge A Murphy HJ Harer MW Jetton JG: Fluid balance in the critically ill child section: “How Bad Is Fluid in Neonates?.” Front Pediatr. 9: 651458, 2021. 33959572 10.3389/fped.2021.651458 PMC8093499

[70] Chen H Yu R-G Yin N-N Zhou J-X: Combination of extracorporeal membrane oxygenation and continuous renal replacement therapy in critically ill patients: A systematic review. Crit Care. 18: 675, 2014. 25482187 10.1186/s13054-014-0675-x PMC4277651

[71] Gorga SM Sahay RD Askenazi DJ: Fluid overload and fluid removal in pediatric patients on extracorporeal membrane oxygenation requiring continuous renal replacement therapy: A multicenter retrospective cohort study. Pediatr Nephrol. 35: 871–882, 2020. 31953749 10.1007/s00467-019-04468-4 PMC7517652

[72] Ostermann M Connor M Jr Kashani K: Continuous renal replacement therapy during extracorporeal membrane oxygenation: Why, when and how? Curr Opin Crit Care. 24: 493–503, 2018. 30325343 10.1097/MCC.0000000000000559

[73] Seczynska B Krolikowski W Nowak I Jankowski M Szuldrzynski K Szczeklik W: Continuous renal replacement therapy during extracorporeal membrane oxygenation in patients treated in medical intensive care unit: Technical considerations. Ther Apher Dial. 18: 523–534, 2014. 25195931 10.1111/1744-9987.12188

[74] de Tymowski C Augustin P Houissa H: CRRT connected to ECMO: Managing high pressures. ASAIO J. 63: 48–52, 2017. 27660903 10.1097/MAT.0000000000000441

[75] Martins Costa A Halfwerk F Wiegmann B Neidlin M Arens J: Trends, advantages and disadvantages in combined extracorporeal lung and kidney support from a technical point of view. Front Med Technol. 4: 909990, 2022. 35800469 10.3389/fmedt.2022.909990 PMC9255675

[76] Martins Costa A Halfwerk FR Thiel J-N Wiegmann B Neidlin M Arens J: Effect of hollow fiber configuration and replacement on the gas exchange performance of artificial membrane lungs. J Membr Sci. 680: 121742, 2023.

[77] Martins Costa A Halfwerk F Thiel J-N Wiegmann B Neidlin M Arens J: Influence of utilizing hemodialysis membranes outside-in on solute clearance and filtration efficiency—One step towards a novel combined lung and kidney support device. J Membr Sci. 698: 122575, 2024.

[78] Gkiougki E Chatziioannidis I Pouliakis A Iacovidou N: Periviable birth: A review of ethical considerations. Hippokratia. 25: 1–7, 2021. 35221649 PMC8877922

[79] Smith LJ McKay KO van Asperen PP Selvadurai H Fitzgerald DA: Normal development of the lung and premature birth. Paediatr Respir Rev. 11: 135–142, 2010. 20692626 10.1016/j.prrv.2009.12.006

[80] Kornhauser Cerar L Lucovnik M: Ethical dilemmas in neonatal care at the limit of viability. Children (Basel). 10: 784, 2023. 37238331 10.3390/children10050784 PMC10217697

[81] van Haren JS van der Hout-van der Jagt MB Meijer N: Simulation-based development: Shaping clinical procedures for extra-uterine life support technology. Adv Simul (Lond). 8: 29, 2023. 38042828 10.1186/s41077-023-00267-y PMC10693037

[82] Feldman R Eidelman AI: Skin-to-skin contact (Kangaroo Care) accelerates autonomic and neurobehavioural maturation in preterm infants. Dev Med Child Neurol. 45: 274–281, 2003. 12647930 10.1017/s0012162203000525

[83] Badr HA Zauszniewski JA: Kangaroo care and postpartum depression: The role of oxytocin. Int J Nurs Sci. 4: 179–183, 2017. 31406740 10.1016/j.ijnss.2017.01.001 PMC6626107

[84] Flacking R Lehtonen L Thomson G; Separation and Closeness Experiences in the Neonatal Environment (SCENE) group: Closeness and separation in neonatal intensive care. Acta Paediatr. 101: 1032–1037, 2012. 22812674 10.1111/j.1651-2227.2012.02787.x PMC3468719

[85] Bagby K Bowen S: Kangaroo care increases breastfeeding rates. J Obstet Gynecol Neonatal Nurs. 41: S49, 2012.

[86] van der Hout-van der Jagt MB Verweij EJT Andriessen P: Interprofessional consensus regarding design requirements for liquid-based perinatal life support (PLS) technology. Front Pediatr. 9: 793531, 2021. 35127593 10.3389/fped.2021.793531 PMC8809135

[87] Verweij EJ De Proost L van Laar JOEH: Ethical development of artificial amniotic sac and placenta technology: a roadmap. Front Pediatr. 9: 793308, 2021. 34956991 10.3389/fped.2021.793308 PMC8694243

[88] UNICEF: UN Convention on the Rights of the Child, 1990.

[89] De Bie FR Kim SD Bose SK: Ethics considerations regarding artificial womb technology for the fetonate. Am J Bioeth. 23: 67–78, 2023. 10.1080/15265161.2022.2048738 35362359

[90] Geurtzen R Wilkinson DJC: Incorporating parental values in complex paediatric and perinatal decisions. Lancet Child Adolesc Health. 8: 225–235, 2024. 38219752 10.1016/S2352-4642(23)00267-5

[91] EFCNI: European Standards of Care for Newborn Health: Rights of infants, parents, and families in difficult decisions., 2018.

[92] Flake AW De Bie FR Munson DA Feudtner C: The artificial placenta and EXTEND technologies: One of these things is not like the other. J Perinatol. 43: 1343–1348, 2023. 37393398 10.1038/s41372-023-01716-2

[93] Allmark P Spedding M: Clinical trials in neonates: Ethical issues. Semin Fetal Neonatal Med. 12: 318–323, 2007. 17350356 10.1016/j.siny.2007.01.023

[94] Mason SA Allmark PJ: Obtaining informed consent to neonatal randomised controlled trials: Interviews with parents and clinicians in the Euricon study. Lancet. 356: 2045–2051, 2000. 11145490 10.1016/s0140-6736(00)03401-2

[95] Neyro V Elie V Thiele N Jacqz-Aigrain E: Clinical trials in neonates: How to optimise informed consent and decision making? a European Delphi survey of parent representatives and clinicians. PLoS One. 13: e0198097, 2018. 29897934 10.1371/journal.pone.0198097 PMC5999079

[96] Heyer J Schubert F Lunemann M: CARD8: A gas-exchange-area-adjustable oxygenator for extremely preterm infants. ASAIO J. 69: 59–59, 2023.36583771

[97] Barton R Ignjatovic V Monagle P: Anticoagulation during ECMO in neonatal and paediatric patients. Thromb Res. 173: 172–177, 2019. 29779622 10.1016/j.thromres.2018.05.009

[98] Fallon BP Lautner-Csorba O Major TC: Extracorporeal life support without systemic anticoagulation: A nitric oxide-based non-thrombogenic circuit for the artificial placenta in an ovine model. Pediatr Res. 95: 93–101, 2023. 37087539 10.1038/s41390-023-02605-2 PMC10600655

[99] Spencer BL Mychaliska GB: Milestones for clinical translation of the artificial placenta. Semin Fetal Neonatal Med. 27: 101408, 2022. 36437184 10.1016/j.siny.2022.101408

