# Supplementary material for: Artificial Placenta and Artificial Womb Technologies for Lung and Kidney Failure: A Holistic Perspective
Source: ASAIO J. 2025 Apr 25;71(7):519–27. doi: 10.1097/MAT.0000000000002443 (PMC12199802; doi:10.1097/MAT.0000000000002443)
Supplement: Supplementary file 1 [file mat-71-0519-s001.pdf]

Supplement A: Comparison table of system features for artificial placenta, fluid filled lung, and artificial womb systems.

	Artificial Placenta System			Fluid filled lung system	Artificial Womb System			
**Group**	McMaster (Hamilton, Ontario, Canada)[1,2]	RWTH Aachen University, (Aachen, Germany)[3]	ArtPlac consortium (Germany, Ireland, Netherlands, Portugal, Sweden, and Canada)[4]	University of Michigan (Ann Arbor, MI, USA)[5-7*]	University of Western Australia and Tohoku University Hospital (Perth, Australia, Sendai, Japan)[8-13]	Children's Hospital of Philadelphia (Philadelphia, PA, USA)[14-16]	Perinatal Life Support consortium (Eindhoven, Netherlands, Aachen, Germany, and Milano, Italy) [17,18]	University of Barcelona (Barcelona, Spain)[19]
**Most recent publication year**	2020[1]	2014[20]	2024[4]	2020[5]	2020[8]	2019[15]	2024[18]	2023[19]
**Model name**	Microfluidic oxygenator	NeonatOx	Artificial Placenta (ArtPlac) Lung Kidney Assist Device	VV preemie ECLS (M-lung)	Ex-vivo uterine environment (EVE)	EXTra-uterine environment for neonatal development (EXTEND)	Perinatal Life Support (PLS)	Artificial uterus system
**State of technology**	In-vitro tested	In-vivo tested	Under development	In-vivo tested	In-vivo tested	In-vivo tested	In-vitro tested	In-vivo tested
**Oxygenator type**	Microfluidic	Hollow fiber	Hollow fiber, Microfluidic	Hollow fiber	Hollow fiber	Hollow fiber	-	Hollow fiber
**Oxygenator model**	Custom	Custom	Custom	Commercial (Medos HiLite)	Commercial (Nipro Corporation)	Commercial (Maquet Quadrox-i Neonatal & Pediatric)	-	Commercial (Maquet Quadrox-i Neonatal & Pediatric)
**Environment**	Incubator	Incubator	Incubator	Incubator	Amniotic fluidic environment	Amniotic fluidic environment	Amniotic fluidic environment	Amniotic fluidic environment
**Pump type**	Fetal heart	Fetal heart	Fetal heart	M-pump (non-occlusive roller pump)	Fetal heart	Fetal heart	Fetal Heart	Fetal heart
**Circuit configuration**	Arteriovenous	Arteriovenous	Arteriovenous	Venovenous	Arteriovenous	Arteriovenous	Arteriovenous	Arteriovenous
**Umbilical vessel use**	Yes	Yes	Yes	Yes (vein only)	Yes	Yes	Yes	Yes
**Drainage cannula**	Umbilical arteries	Umbilical arteries	Umbilical arteries	Internal jugular vein	Umbilical arteries	Umbilical arteries	Umbilical arteries	Umbilical arteries
**Reinfusion cannula**	Umbilical vein	Umbilical vein	Umbilical vein	Umbilical vein / Internal jugular vein	Umbilical vein	Umbilical vein	Umbilical vein	Umbilical vein

**Mode of delivery**	C-section & natural (envisioned)	C-section<br><br>(applied in vivo)	C-section & natural<br><br>(envisioned)	C-section<br><br>(applied in vivo)	C-section<br><br>(applied in vivo)	C-section<br><br>(applied in vivo)	C-section & natural[21]<br><br>(envisioned)	C-section<br><br>(applied in vivo)
**Airway management**	Aerated lungs	Aerated lungs	Aerated lungs	Fluid filled lungs	Fluid filled lungs	Fluid filled lungs	Fluid filled lungs	Fluid filled lungs
**Initiation of treatment**	After CPAP failure	After CPAP failure	After CPAP failure	During EXIT procedure	During EXIT procedure	During EXIT procedure	During EXIT procedure	During EXIT procedure
**Monitoring before treatment possible**	Yes	Yes	Yes	No	No	No	No	No
**Respiratory support**	Yes	Yes	Yes	Yes	Yes	Yes	Yes	Yes
**Renal support**	No	No	Yes	No	No	No	No	No
**Family centered care possible**	Yes	Yes	Yes	Yes	No	No	No	No
**Fetal physiology maintained**	Only umbilical vein, umbilical arteries and ductus venosus	No	Only umbilical vein, umbilical arteries and ductus venosus	Yes	Yes	Yes	Yes	Yes

Abbreviations – VV: venovenous, ECLS: Extra Corporeal Life Support, C-section: caesarean section, EXIT: ex-utero intrapartum therapy, CPAP: continuous positive airway pressure

*This group is also working on a microfluidic artificial placenta, but the device has not been tested in vivo, yet.

References

1. Mohammadhossein Dabaghi NR, Neda Saraei, Rupesh Kumar Mahendran, Gerhard Fusch, Anthony K. C. Chan, John L. Brash, Christoph Fusch, and Ponnambalam Ravi Selvaganapathy: Miniaturization of Artificial Lungs toward Portability. 2020 doi: 10.1002/admt.202000136.

2. Dabaghi M, Fusch G, Saraei N, *et al*: An artificial placenta type microfluidic blood oxygenator with double-sided gas transfer microchannels and its integration as a neonatal lung assist device. *Biomicrofluidics* 12 (4): 044101, 2018 doi: 10.1063/1.5034791.

3. Arens J, Schoberer M, Lohr A*, et al*: NeonatOx: a pumpless extracorporeal lung support for premature neonates. *Artif Organs* 35 (11): 997-1001, 2011 doi: 10.1111/j.1525-1594.2011.01324.x.

4. ArtPlac: Artificial Placenta (ArtPlac) - Miniaturized Integrated Lung and Kidney Support for Critically Ill Newborns. Available at: https://artplac.eu/, 2024.

5. Kading JC, Langley MW, Lautner G*, et al*: Tidal Flow Perfusion for the Artificial Placenta: A Paradigm Shift. *Asaio J* 66 (7): 796-802, 2020 doi: 10.1097/MAT.0000000000001077.

6. Bryner B, Gray B, Perkins E*, et al*: An extracorporeal artificial placenta supports extremely premature lambs for 1 week. *J Pediatr Surg* 50 (1): 44-9, 2015 doi: 10.1016/j.jpedsurg.2014.10.028.

7. Reoma JL, Rojas A, Kim AC*, et al*: Development of an artificial placenta I: pumpless arterio-venous extracorporeal life support in a neonatal sheep model. *J Pediatr Surg* 44 (1): 53-9, 2009 doi: 10.1016/j.jpedsurg.2008.10.009.

8. Usuda H, Watanabe S, Saito M*, et al*: Successful use of an artificial placenta-based life support system to treat extremely preterm ovine fetuses compromised by intrauterine inflammation. *Am J Obstet Gynecol* 223 (5): 755 e1-755 e20, 2020 doi: 10.1016/j.ajog.2020.04.036.

9. Usuda H, Watanabe S, Saito M*, et al*: Successful use of an artificial placenta to support extremely preterm ovine fetuses at the border of viability. *Am J Obstet Gynecol* 221 (1): 69 e1-69 e17, 2019 doi: 10.1016/j.ajog.2019.03.001.

10. Miura Y, Usuda H, Watanabe S, *et al*: Stable Control of Physiological Parameters, But Not Infection, in Preterm Lambs Maintained on Ex Vivo Uterine Environment Therapy. *Artif Organs* 41 (10): 959-968, 2017 doi: 10.1111/aor.12974.

11. Miura Y, Matsuda T, Usuda H, *et al*: A Parallelized Pumpless Artificial Placenta System Significantly Prolonged Survival Time in a Preterm Lamb Model. *Artif Organs* 40 (5): E61-8, 2016 doi: 10.1111/aor.12656.

12. Miura Y, Saito M, Usuda H, *et al*: Ex-Vivo Uterine Environment (EVE) Therapy Induced Limited Fetal Inflammation in a Premature Lamb Model. *PLoS One* 10 (10): e0140701, 2015 doi: 10.1371/journal.pone.0140701.

13. Miura Y, Matsuda T, Funakubo A, *et al*: Novel modification of an artificial placenta: pumpless arteriovenous extracorporeal life support in a premature lamb model. *Pediatr Res* 72 (5): 490-4, 2012 doi: 10.1038/pr.2012.108.

14. Partridge EA, Davey MG, Hornick MA, Flake AW: An EXTrauterine environment for neonatal development: EXTENDING fetal physiology beyond the womb. *Seminars in Fetal and Neonatal Medicine* [Review] 22 (6): 404-409, 2017 doi: 10.1016/j.siny.2017.04.006.

15. Hornick MA, Mejaddam AY, McGovern PE, *et al*: Technical feasibility of umbilical cannulation in midgestation lambs supported by the EXTra-uterine Environment for Neonatal Development (EXTEND). *Artif Organs* 43 (12): 1154-1161, 2019 doi: 10.1111/aor.13524.

16. Hornick MA, Davey MG, Partridge EA*, et al*: Umbilical cannulation optimizes circuit flows in premature lambs supported by the EXTra-uterine Environment for Neonatal Development (EXTEND). *Journal of Physiology* [Article] 596 (9): 1575-1585, 2018 doi: 10.1113/JP275367.

17. Eindhoven TU: INCUBATION SYSTEM FOR LIQUID - BASED INCUBATION OF PREMATURELY BORN INFANTS, in Eindhoven TU (ed), *Patent*, Netherlands, 2022.

18. van Haren JS, Delbressine FLM, Schoberer M*, et al*: Transferring an extremely premature infant to an extra-uterine life support system: a prospective view on the obstetric procedure. *Front Pediatr* 12: 1360111, 2024 doi: 10.3389/fped.2024.1360111.

19. Eixarch E, Illa M, Fucho R*, et al*: An Artificial Placenta Experimental System in Sheep: Critical Issues for Successful Transition and Survival up to One Week. *Biomedicines* [Article] 11 (3), 2023 doi: 10.3390/biomedicines11030702.

20. Schoberer M, Arens J, Erben A*, et al*: Miniaturization: the clue to clinical application of the artificial placenta. *Artif Organs* 38 (3): 208-14, 2014 doi: 10.1111/aor.12146.

21. van Haren JS, van der Hout-van der Jagt MB, Meijer N*, et al*: Simulation-based development: shaping clinical procedures for extra-uterine life support technology. *Adv Simul (Lond)* 8 (1): 29, 2023 doi: 10.1186/s41077-023-00267-y.
